# Immunomodulatory Gene-Splicing Dysregulation in Tumorigenesis: Unmasking the Complexity

**DOI:** 10.3390/molecules28165984

**Published:** 2023-08-10

**Authors:** Lorraine Tshegofatso Maebele, Thanyani Victor Mulaudzi, Madhavan Yasasve, Zodwa Dlamini, Botle Precious Damane

**Affiliations:** 1 Department of Surgery, Steve Biko Academic Hospital, University of Pretoria, Hatfield 0028, South Africa; 2 Department of Oral Medicine and Radiology, Sri Ramachandra Institute of Higher Education and Research, Chennai 600116, India; 3 SAMRC Precision Oncology Research Unit (PORU), DSI/NRF SARChI Chair in Precision Oncology and Cancer Prevention (POCP), Pan African Cancer Research Institute (PACRI), University of Pretoria, Hatfield 0028, South Africa

**Keywords:** alternative splicing, isoforms, immune checkpoints, immunomodulatory proteins, splicing factors

## Abstract

Cancer is a global health concern with rising incidence, morbidity, and mortality. The interaction between the tumor and immune cells within the tumor microenvironment is facilitated by signaling pathways driven by immunomodulatory proteins. Alternative splicing regulates the production of multiple immunomodulatory proteins with diverse functionality from a single mRNA transcript. Splicing factors are pivotal in modulating alternative splicing processes but are also subject to regulation. The dysregulation of alternative splicing may result from splicing factor (SF) abnormal expression levels and mutations in the cis and trans-acting elements and small nuclear RNA (snRNA) molecules. Aberrant splicing may generate abnormal mRNA transcripts encoding isoforms with altered functions that contribute to tumorigenesis or cancer progression. This review uncovers the complexity of immunomodulatory genes splicing dysregulation in oncogenesis. Identifying specific immunomodulatory splicing isoforms that contribute to cancer could be utilized to improve current immunotherapeutic drugs or develop novel therapeutic interventions for cancer.

## 1. Introduction

Cancer is one of the leading causes of mortality, with an estimation of 10 million deaths worldwide per year [1]. The disease burden generated by cancer globally is tremendous and projected to increase in the next two decades [2]. Precursor mRNA splicing has a significant contribution towards tumor initiation and progression [1]. This is because alternative splicing is dysregulated in almost all cancer types [3]. Alternative splicing is a process of producing mature mRNA molecules from pre-mRNA catalyzed by a spliceosome complex. Aberrant splicing may result from small nuclear RNA (snRNA) and splicing factor (SF) mutations and SF abnormal expression levels [4]. The dysregulated alternative splicing process generates abnormal mRNA transcripts encoding aberrant protein isoforms with altered functions resulting in cancer development [5]. Under normal physiological conditions, the alternative splicing of pre-mRNA from a single gene is aimed at promoting transcriptome and proteome diversity [6], but in cancer, it contributes to tumor heterogeneity and cellular plasticity [3]. This equips tumor cells with dynamic and adaptable phenotypic traits allowing them to escape an immune response [7].

The immune system is a complex and dynamic physiological system functioning via the integration of a network of connections consisting of cells and molecules [8]. An innate immunity non-specifically detects the presence of any abnormality, such as tumors, while adaptive immunity responds specifically to tumor antigens [9]. Thus, the interaction between innate and adaptive immune responses is pivotal in elucidating an effective anti-tumor immunity [10]. A strong immune response can be generated without damaging host organs by maintaining a balance between stimulatory or inhibitory signals, which are tightly regulated by immunomodulatory molecules [11,12]. The expression levels and the interaction between immunomodulatory protein receptors with their ligands are critical for the regulation of an immune response [13]. The dysregulation of an immune response is associated with oncogenesis [14,15]. Studies have indicated the implication of alternative splicing in immune dysregulation [16] and also the presence of abnormal isoforms and their aberrant expression levels as cancer hallmarks [17]. This review unmasks the complexity of immunomodulatory genes splicing dysregulation in carcinogenesis. Understanding immunomodulatory splicing isoforms in cancers is of great importance for uncovering their role in mechanisms of tumor immune escape and suppression [18]. This knowledge can be applied in the development of novel, selective and highly potent anti-cancer therapies [19]. 

## 2. Methodology

A literature search was carried out on Scopus, Web of Science, Google Scholar and PubMed databases. Keywords and phrases used for search included ‘Immunomodulatory genes’, ‘alternative splicing’, ‘Immunomodulatory genes’ AND ‘alternative splicing’, and ‘aberrant splicing isoforms AND cancer’. For specific isoforms reviewed, we searched using the name of the immunomodulatory isoforms, for example, ‘CTLA-4 isoforms AND cancer’, and lastly searched the ‘immunomodulatory isoform and splicing factors’ for all isoforms, for example, ‘sCTLA-4 and splicing factors’. A broad search on all the cancers was performed, and no period was specified during the search. A total of 243 articles were retrieved. Articles were reviewed based on their titles and abstracts to evaluate their relevance to the topic, and those with no relevance to immunomodulatory genes splicing dysregulation in cancer were excluded. The number of articles that passed this stage was 124, and they were further analyzed to structure the outline of the topic. 

## 3. Alternative Splicing Mechanism

**Figure 1:**
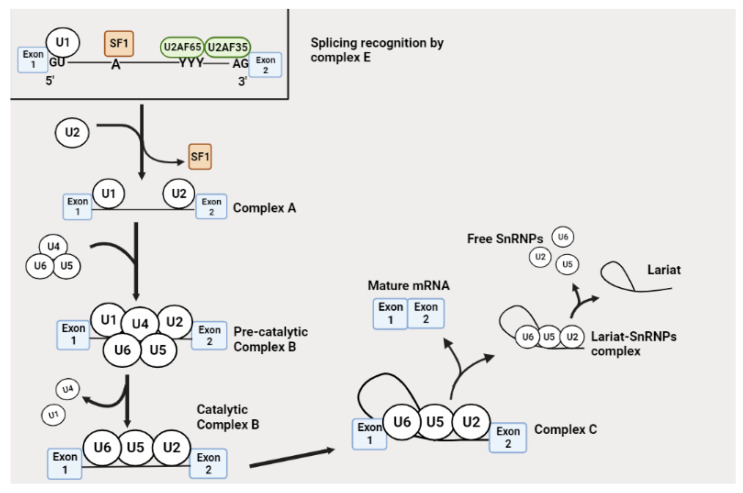
The splicing mechanism. The elements of complex E such as U1, U2AF65, U2AF35 and SF1 initiate splicing by binding to the 5′ splice site, PY-tract, 3′ splice site and the BP, respectively. The recruitment of U2 replaces SF1 forming complex A. Then, the U4/U5/U6 tri-SnRNP interacts with complex A to form an inactive complex B, which then undergoes conformational and catalytic activation by releasing U1 and U4 to form a catalytic complex B. The active complex B catalyzes the cleavage of the intron at the 5′ end generating complex C. This complex finalizes the removal of the intron at the 3′ site to release the lariat/U2/U5/U6 complex and the ligation of exons to generate a mature RNA. The lariat/U2/U5/U6 complex further dissociates into a lariat and frees U2, U5 and U6, which can be re-used in another splicing process. Image created with BioRender.com, accessed on 20 May 2023.

Spliceosome assembly initiates by the recognition of the 5′ splice site through the binding of U1 snRNP to the precursor-mRNA. The U2 Auxiliary Factor (U2AF) subunits, namely U2AF35 and U2AF65, interact with the 3′ splice site and the polypyrimidine tract (PY-tract), respectively. Then, the bridging SF1 attaches to branch point A (BP) and connects U1 and U2AF subunits to form a complex E. This complex triggers the recruitment of U2, which replaces the SF1 at the branch point to generate complex A [20,21,22]. U2AF65 and U2AF35 are reported to dissociate at the later stages of splicing [22]. The U4/U5/U6 trimer binds to U1 and U2 forming an inactive complex B, which undergoes compositional and conformational rearrangements. U1 and U4 are released to form a catalytic complex B. The active complex B catalyzes the excision of the intron at the 5′-end and the formation of a lariat generating complex C [21,22]. This complex undergoes rearrangements in the spliceosome′s ribonucleoproteins network. Subsequently, complex C catalyzes the excision of the 3′end, releasing the lariat/U2/U5/U6 complex and a combination of the exons. The lariat/U2/U5/U6 complex dissociates. U2, U5 and U6 can then be used in the future splicing process [20,21,22]. The splicing mechanism is illustrated in Figure 1.

## 4. Alternative Splicing in Immunomodulatory Genes

### 4.1. Cytotoxic T Lymphocyte-Associated Protein 4 (CTLA-4)

CTLA-4 also referred to as CD152 [23] is a co-inhibitory receptor expressed on the plasma membrane of activated cytotoxic T cells, helper T cells, T regs, memory T cells, natural killer cells [24], monocytes, granulocytes, B cells, skeletal muscle cells, placental fibroblasts [23] and in lung, bladder, ovarian, colon, renal, breast, uterine, rhabdomyosarcoma, melanoma and neuroblastoma cancer cell lines [25]. CTLA-4 is immunosuppressive, unlike its homolog CD28, as it can prevent antigen presentation and naive helper CD4+ T cell activation. It mediates immunosuppression by scavenging CD80 or CD86 through high affinity binding than CD28 and via trans-endocytosis in antigen-presenting cells (APCs) [26]. CTLA-4 is primarily localized intracellularly, and its translocation to the immunological synapse is dependent on the stimulatory signals caused by major histocompatibility complex (MHC) and T cell receptor (TCR) and CD28-CD80/86 binding. The exocytosis of vesicles containing CTLA-4 at the plasma membrane surface is dependent on the TCR signaling strength [27].

**Figure 2:**
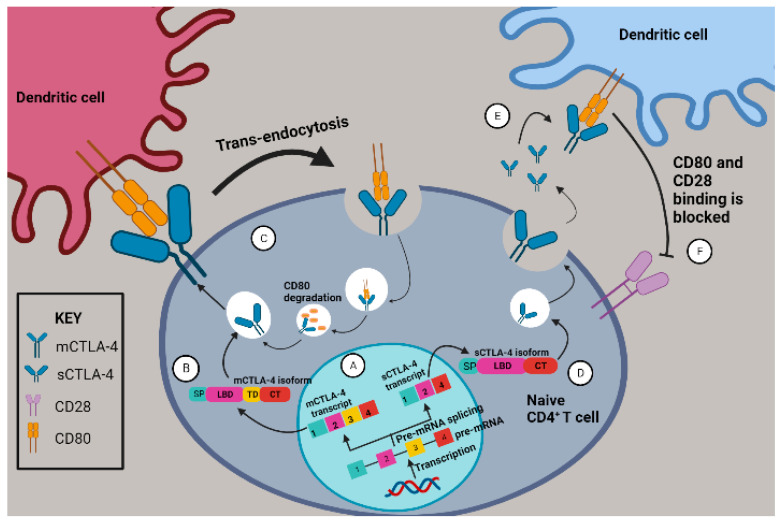
An illustration of CTLA-4 isoforms mCTLA-4 and sCTLA-4 and their inhibitory roles during naive T cell activation by an antigen-presenting cell. (**A**) The alternative splicing of *CTLA-4* pre-mRNA transcript generates *mCTLA-4* and *sCTLA-4* splice variants. (**B**) *mCTLA-4* is translated into mCTLA-4 isoform. (**C**)The functional mCTLA-4 protein molecule can be transported to the immunological synapse via intracellular vesicles. The cell surface mCTLA-4 interacts with CD80 and causes trans-endocytosis, whereby the mCTLA-4-CD80 complex is engulfed into a lysosome at which CD80 is degraded. mCTLA-4 is recycled back to the cell surface during another CD28 and CD80 interaction. (**D**) *sCTLA-4* is translated into sCTLA-4 isoform. (**E**) The functional sCTLA-4 protein lacks the membrane binding domain, which is encoded by exon 3. sCTLA-4 is released into the cytosol owing to the lack of transmembrane domain. (**F**) Upon release, the soluble CTLA-4 isoform binds to the membrane-bound CD80 and blocks the CD80-CD28 interaction resulting in a co-inhibition. Image created with BioRender.com, accessed on 15 May 2023.

*CTLA-4* gene comprises four exons with exon 1 encoding a signaling peptide (SP), exon 2 encoding the ligand binding domain (LBD), exon 3 encoding the transmembrane domain (TD) and exon 4 encoding the cytoplasmic tail (CT) [28]. CTLA-4 has two major isoforms generated by alternative splicing, namely sCTLA-4 and mCTLA-4, which are expressed in tumor cells and implicated in cancer immunosurveillance escape. They both bind to similar ligands namely CD80 and CD86 with greater affinity than CD28. The sCTLA-4 lacks exon 3 that codes for the transmembrane domain [29], binds to CD80 and blocks its interaction with CD28 in APCs consequently inhibiting T cells activation [30]. mCTLA-4 depletes CD80 and CD86 from the APCs cell surface through trans-endocytosis, as illustrated in Figure 2 [29]. Serum sCTLA-4 is associated with drug response and better patient survival in melanoma patients treated with ipilimumab (Table 1) [31]. sCTLA-4 levels can function as a predictor of disease recurrence in hepatocellular carcinoma (HCC) patients treated with radiofrequency ablation [32]. Selective sCTLA-4 blockade inhibits metastatic melanoma in mice suggesting its involvement in metastasis [33]. sCTLA-4 was shown to be associated with shorter survival in glioma patients (Table 1) [34]. Furthermore, sCTLA-4 was found to be a potential marker of disease progression in acute lymphoblastic leukemia patients as the overexpression of sCTLA-4 positively correlated with the percentage of leukemic B cells [35].

### 4.2. Programmed Death 1 (PD-1)

PD-1 belongs to the CD28/B7 subgroup of the immunoglobulin (Ig) superfamily, which is localized on the cell surface of the activated B cells, monocytes and T cells. PD-1 inhibits T-cell proliferation and survival when bound to programmed death ligand 1 (PD-L1) or programmed death ligand 2 (PD-L2) by blocking the IL-2, IFN-γ and TNF-α production. It is important for preventing autoimmunity and maintaining self-tolerance by inhibiting T-cell activation during the effector phase [27,46]. Various studies have shown that PD-1 deficiency in mice models generates autoimmunity exhibited by the occurrence of conditions in mice such as lupus-like arthritis, glomerulonephritis, fetal dilated cardiomyopathy [47] and fatal myocarditis [48]. The high expression of PD-1 promotes tumor immune surveillance escape and cancer progression, for example, in colorectal cancer. Furthermore, high PD-1 levels correlate with a poor prognosis in esophageal cancer, primary central nervous system lymphoma (PCNSL) [49] and cervical adenocarcinoma [50]. The blockade of PD-1 enhances NK cell activity and antibody secretion by activating the PD-1-expressing B-cells in addition to upregulating effector T cell functions in tissues and TME [47]. 

**Figure 3:**
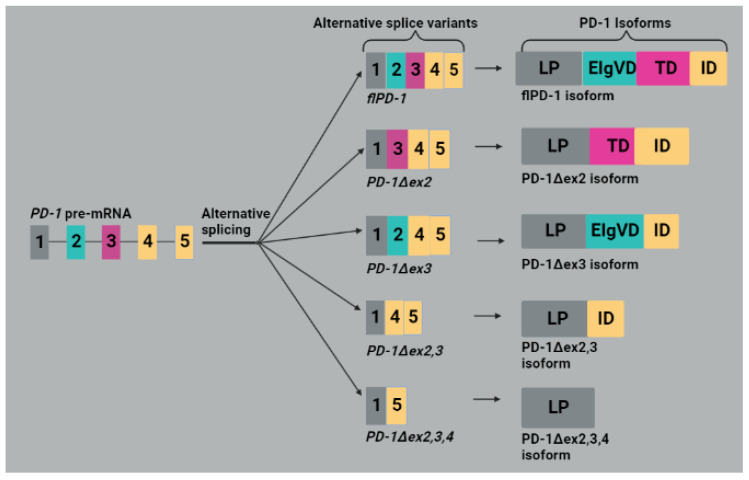
An illustration of *PD-1* splice variants generated by alternative splicing and their isoforms. Alternative splicing of *PD-1* pre-mRNA generates five spice variants, namely *fIPD-1, PD-1Δex2, PD-1Δex3, PD-1Δex2,3* and *PD-1Δex2,3,4*, of which are translated into fIPD-1, PD-1Δex2, PD-1Δex3, PD-1Δex2,3 and PD-1Δex2,3,4 isoforms, respectively. fIPD-1 is a full-length isoform containing LP, EIgVD, TD and ID domains. PD-1Δex2 lacks EIgVD domain, whereas PD-1Δex3 lacks only the TD domain. PD-1Δex2,3 isoform consists of the LP and ID domains, and PD-1Δex2,3,4 consists of the LP domain only. Image created with BioRender.com, accessed on 30 June 2023.

Five mRNA splice variants, namely *PD-1Δex2, PD-1Δex3, PD-1Δex2,3, PD-1Δex2,3,4* and *fIPD-1*, were detected from human PBMCs, as shown in Figure 3. The *fIPD-1* is also referred to as a full variant as it contains exons 1 (encoding leader peptide (LP)), 2 (extracellular IgV-like domain (EIgVD)), 3 (transmembrane domain (TD)) and 4 and 5 (intracellular domain (ID)). They are named according to the exons they lack; for example, the number after ex on the name denotes excised exon(s). *PD-1Δex2* lacks exon 2, which coded for the extracellular IgV-like domain meaning that the resulting isoform cannot bind the PD-L1 and PD-L2 ligands. *PD-1Δex3* variant encodes a soluble form of PD-1 (sPD-1), which can easily bind to PD-L1/L2 compared to the membrane-bound PD-1 and interferes with the interaction between PD-1 and PD-L1/2 [46]. The sPD-1 was shown to attenuate the inhibition of T cell activity in a TME and consequently restore anti-tumor immunity [51]. The *PD-1Δex2,3* variant is suggested to not encode an apparent functional putative protein as it lacks exons for both the intramembrane and the ligand binding domains. The translation of *PD-1Δex2,3,4* can create a premature STOP codon in exon 5 and generate a protein lacking the extracellular IgV-like, cytosolic and transmembrane domains (Figure 3) [46].

### 4.3. Programmed Death Ligand 1 (PD-L1)

PD-L1 is a co-inhibitory molecule expressed on the cell surface of B cells, dendritic cells (DCs), natural killer cells, macrophages, T cells, MDSCs, endothelial, epithelial and tumor cells. The overexpression of PD-L1 is strongly linked to advanced disease and unfavorable prognosis in the bladder, breast, pancreatic, ovarian, melanoma, kidney, gastric and liver cancers [52]. PD-L1 facilitates epithelial–mesenchymal transition (EMT) in breast tumor stem cells and is linked to metastatic disease and unfavorable clinical outcomes in colorectal cancer. The expression of PD-L1 in ovarian cancer cells was shown to be upregulated by the presence of IFN-γ [53]. *PD-L1/CD274* gene consists of seven exons with exon 1 coding for the 5′ untranslated region (5′ UTR), exon 2 encoding signaling peptide (SP), exon 3 encoding the IgV-like domain (IgVD), exon 4 encoding IgC-like domain (IgCD), exon 5 encoding the transmembrane domain (TD), exon 6 encoding intracellular domain (ID) and exon 7 encoding a portion of the ID and a 3′ untranslated region (3′UTR) [54].

**Figure 4:**
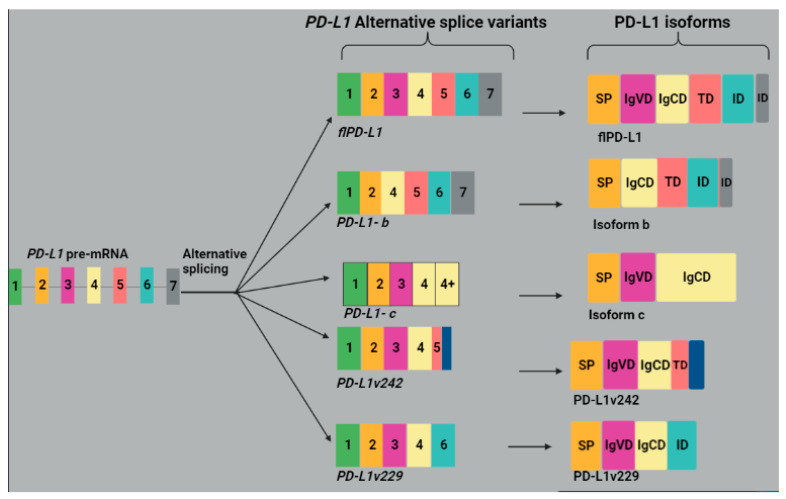
An illustration of PD-L1 splice variants generated by alternative splicing and their isoforms. Alternative splicing of *PD-L1* pre-mRNA generates splice variants, namely *flPD-L1, PD-L1b, PD-L1c, PD-L1v229* and *PD-L1v242*. The *flPD-L1* is a full-length transcript with all exons from 1 to 7, and its resulting isoform contains all the domains, namely SP, IgVD, IgCD, TD and ID. Exon 1 encodes 5′ UTR, and exon 7 encodes a portion of ID and a 3′ UTR. The transcript variant *PD-L1b* lacks exon 3; hence, the resulting protein does not have the IgVD. *PD-L1c* lacks exons 5, 6 and 7, but its resulting protein has an extended C-terminal. PD-L1v242 and PD-L1v229 are soluble isoforms, both containing SP, IgVD and IgCD domains. PD-L1v229 also contains the ID, and PD-L1v242 has a TD with deletion and extra amino acids at its C-terminal. Image created with BioRender.com, accessed on 30 June 2023.

The alternative splicing of the *PD-L1* gene in colorectal cancer (CRC) generated isoforms a, b and c. Isoform a is considered a full-length isoform containing all exons from 1 to 7. Isoform b lacks exon 3 and was shown to potently inhibit T cell function more than isoforms a and c and promoted tumor cell immune escape. Furthermore, isoform b was shown to be correlated with an unfavorable prognosis and survival in colorectal cancer patients. Isoform c is a secreted form of PD-L1 lacking the membrane-binding and intracellular domains (Figure 4), capable of binding PD-1 and downregulating T-cell activity. It is implicated in the growth of tumors and metastasis; thus, it is considered a prognostic marker in CRC [55]. Hassounah et al. [56] detected another sPD-L1 protein capable of binding PD-1 and downregulating IL-2 and IFN-γ production in primary T cells. Four soluble PD-L1 isoforms, namely PD-L1-1, PD-L1-3, PD-L1-9 and PD-L1-12, have been detected in melanoma cancer cells and are formed by the presence of a stop codon before the transmembrane domain. They are associated with disease progression in melanoma patients receiving immune checkpoint blockade treatment (Table 1) [37]. Gong and colleagues [38] have detected five PD-L1 splice variants from NSCLC patients who relapsed from anti-PD-L1 therapy, consisting of a full-length variant encoding a membrane-bound isoform and four variants encoding the soluble form of PD-L1, among which only two were proven to be stable. These stable isoforms, namely PD-L1v229 and PD-L1v242 (Figure 4), were shown to act as decoys and bind to PD-L1 blockade in vitro as a result promoting the PD-L1 and PD-1 interaction, which further suppressed the anti-tumor immunity (Table 1). 

### 4.4. Human Leukocyte Antigen G (HLA-G)

HLA-G has potent co-inhibitory effects on an anti-tumor immune response compared to other immune checkpoint molecules. The interaction between HLA-G with its receptors such as ILT2/CD85j/LILRB1 (ILT2), ILT4/CD85d/LILRB2 (ILT4) and KIR2DL4/CD158d (KIR2DL4) targets B cells, monocytes, NK and T cells; DCs and monocytes; and NK decidual cells, respectively [57]. HLA-G plays a significant role in maintaining fetal–maternal immune tolerance and is used in transplantation [58]. HLA-G inhibits the activity of immune cells via receptor binding, trogocytosis and chemotaxis impairment. The expression of HLA-G promotes tumor immune escape by modulating both the phenotype and function of immune cells leading to immune evasion and metastasis [59]. The conditions in the TME such as the presence of certain cytokines, glucocorticoids, heat shock and hypoxia contribute to the modulation of HLA-G expression [60].

**Figure 5:**
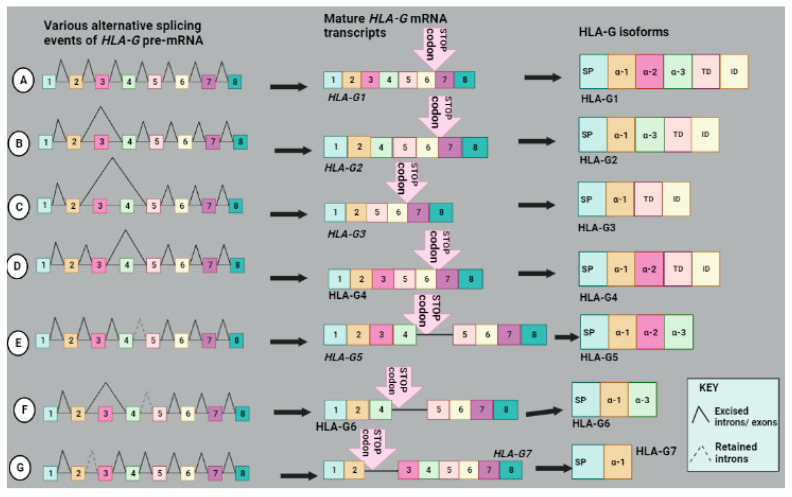
An illustration of *HLA-G* splicing, splice variants and isoforms generated. (**A**) The alternative splicing of membrane-bound HLA-G pre-mRNA molecules generate a stop codon after exon 6; hence, they all do not have domains encoded by exons 7 and 8. The *HLA-G1* variant generated by alternative splicing encodes a full-length HLA-G1 isoform with domains SP, α-1, α-2, α-3, TD and ID encoded by exons 1 to 6. (**B**) The exon 3 skipping generates *HLA-G2* transcript encoding HLA-G2 isoform with domains SP, α-1, α-3, TD and ID. (**C**) Exon 3 and 4 skipping generates *HLA-G3* transcript, which is translated into HLA-G3 isoform consisting of SP, α-1, TD and ID domains. (**D**) The *HLA-G4* splice variant is generated by exon 4 skipping, generating the HLA-G4 isoform without α-3 domain. (**E**) An intron retention between exon 4 and 5 generates an *HLA-G5* transcript with a stop codon after exon 4 resulting in a truncated soluble isoform without TD and ID domains. (**F**) The *HLA-G6* variant is produced through the skipping of exon 3 and the retention of intron 4 and has a stop codon after exon 4. The resulting isoform consists only of SP, α-1 and α-3. (**G**) An intron 2 retention generates an *HLA-G7* splice variant with a stop codon after exon 2 producing an isoform with only the SP and α-1 domains. Image created with BioRender.com, accessed on 30 June 2023.

The alternative splicing of *HLA-G* generates four membrane-bound isoforms, namely HLA-G1 to HLA-G4, and three soluble isoforms, HLA-G5 to HLA-G7, shown in Figure 5. The extracellular protein section of HLA-G1 and HLA-G5 is complete with all three alpha domains, namely α-1, α-2 and α-3, linked to β2 microglobulin (β2M). The other isoforms have different extracellular protein structures and are not linked to β2M. For example, HLA-G2, HLA-G4 and HLA-G7 [61] and HLA-G3 lacks α-2, α-3 or both α-2 and α-3 domains, respectively (Figure 5). The isoforms without α-3 cannot interact with ILT4 or ILT2 receptors [57]. *HLA-G2*, *HLA-G4* and *HLA-G3* are generated by skipping exons 3, 4 and both exons 3 and 4, respectively. HLA-G1 to HLA-G4 isoforms are produced due to the presence of a stop codon in exon 6. The soluble isoforms resulted from the retention of intron 4 by HLA-G5 and HLA-G6 or intron 2 by HLA-G7 that generated a premature STOP codon before the transmembrane domain which led to their secretion. Exon 3 skipping also occurs in *HLA-G6* alternative splicing (Figure 5) [62].

These isoforms are hypothesized to have distinct immunosuppressive functions. The HLA-G5 or -G6 has been shown to create an immunosuppressive environment around the tumor tissue. HLA-G6 has a negative correlation with pathological complete response (pCR) in the HER2+ breast cancer subtype, as the low expression levels of HLA-G6 were consistent with a high pCR rate [61]. M8 melanoma cells secreted the HLA-G5 isoform, which prevented NK cell-mediated cytotoxicity towards the target cell via the impairment of lytic granules polarization [60]. Soluble HLA-G (sHLA-G) detected in blood serum mainly consists of sHLA-G5 and sHLA-G1 (generated from proteolytic cleavage of HLA-G1) [63]. It has been demonstrated to be associated with tumor aggressiveness, tumor-node-metastasis stage, histological type, or a reduced survival period of breast, lung cancer and papillary thyroid carcinoma (PTC) patients [64]. Furthermore, sHLA-G is associated with advanced melanoma stage and tumor load (Table 1) [39]. The soluble HLA-G can serve as a diagnostic marker for distinguishing benign from malignant tumors [60]. HLA-G has great potential for use in overcoming drug resistance by blocking its expression or function [65]. Furthermore, HLA-G might serve as a possible marker for tumor susceptibility to chemotherapy and as a prognostic marker for advanced tumor stage and clinical outcome [60]. 

### 4.5. Simulator of Interferon Genes (STING)

STING is a regulatory protein consisting of four transmembrane domains (TM) located in the endoplasmic reticulum (ER), cytoplasmic ligand binding domain (LBD) and the C-terminal tail or domain (CTD). LBD can homodimerize and undergo conformational changes upon binding by cGAMP. The CTD is responsible for binding and phosphorylation by kinases such as TANK-binding kinase 1 (TBK1) [66]. STING is mostly expressed in innate, adaptive and non-immune cells. The modulation of inflammation by STING begins with the sensing of nucleic acid molecules in the cytosol that might have resulted from viruses, bacteria and dying cells (through phagocytosis) by cGAS, which then uses GTP and ATP to synthesize cGAMP. STING is activated by interacting with cGAMP and then is carried by iRhom and transported from the ER to the Golgi body, where it recruits and activates the TBK1 and the IkB kinase (IKK). TBK1 undergoes autophosphorylation and also phosphorylates STING. Interferon regulatory factor 3 (IRF3) is recruited by STING, phosphorylated by TBK1, and homodimerizes to enter the nucleus, where it activates the transcription of type I Interferons, chemokines and inflammatory cytokines. IKK kinase consisting of the IKKα and IKKβ activates NF-kB, which then enters the nucleus and induces transcription by phosphorylating NF-kB inhibitor (IkB-α) [67,68,69]. In mouse prostate cancer cells, cytosolic DNA triggers the STING pathway and induces anti-cancer immunity [70]. Another piece of evidence proposes that the presence of STING in B16 melanoma cells is associated with the activation of anti-tumor immunity, which inhibits tumor progression [71]. Thus, several studies have proposed that STING activation is a promising strategy in cancer immunotherapy [69]. The downregulation of STING signaling impedes the DNA responses required for generating vital cytokines, including IFN-I, that mediate tissue repair and anti-tumor T cell priming [72].

**Figure 6:**
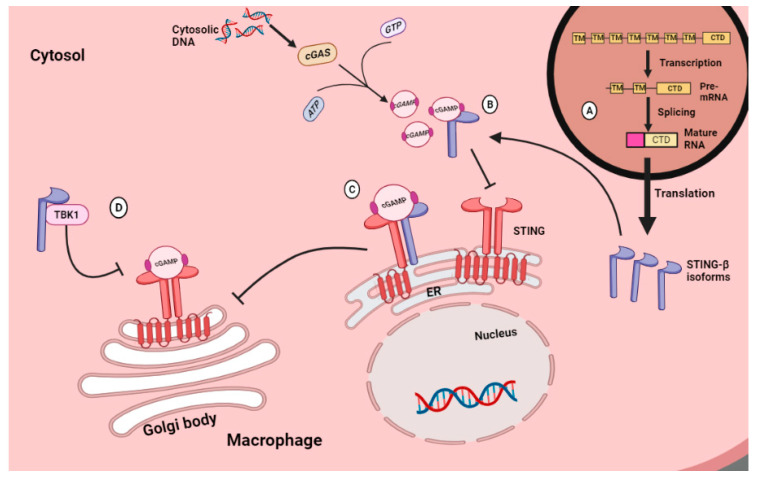
STING signaling pathway inhibition by STING-β in various ways and at various steps. (**A**) The splicing of the *STING* pre-mRNA produces a mature mRNA molecule that can be translated to generate a STING-β protein isoform lacking a membrane binding domain but has a CTD domain that enables its binding to STING ligands. (**B**) The interaction of STING-β with the cGAMP blocks cGAMP binding to the membrane-bound STING isoform resulting in the inhibition of all the upcoming interactions. (**C**) The cGAMP bound STING-β and STING heterodimer complex cannot relocate to the Golgi body due to STING-β’s lack of transmembrane domain. (**D**) STING-β may bind to the free TBK1 and block its interaction with the cGAMP-STING homodimer complex preventing the NF-kB and IRF3 activation and subsequent cytokine production. Image created with BioRender.com, accessed on 17 May 2023.

A plasma membrane-bound isoform (pmSTING) with its C-terminus in the extracellular space has been detected in mouse and human cells. This isoform can be bound by an activated cGAMP to initiate signal transduction after the detection of extracellular DNA [73]. Other STING isoforms have been detected, including MITA-related protein (MRP), truncated isoform 2, truncated isoform 3, STING-β and tSTING-Mini. Isoform 2 lacks exon 4 and 7, isoform 3 lacks exon 7 and contains intron retention after exon 3 [74], and tSTING-Mini is generated from exon skipping of exons from 2 to 5 [75]. They all do not interact with TBK1 due to the lack of CTD, which is a binding domain for TBK1 [74]. Moreover, tSTING-Mini can produce a strong and fast antiviral response by inducing the phosphorylation of tIRF3 without interacting with tTBK1 [75]. MRP is another isoform detected in both mice and humans that inhibits IRF3 activation since it lacks TBK1 binding domain but can activate NF-κB [74,76]. This protein has a dimerization domain and, thus, can form homodimers with itself or heterodimers with STING and both inhibit STING interaction with other proteins such as TBK1 and block IRF3 activation while activating the NF-kB [77]. The other alternatively spliced STING isoform is STING-β. The transcription promoter region is at intron 5 of the STING allele. Therefore, its final transcript lacks exons 1 to 5, and its first exon has an extra ribonucleotide sequence at the 5′ end, causing the product isoform to have more than 25 amino acids at the N-terminus. Despite these extra amino acids, STING-β lacks the transmembrane domain, and thus, it cannot activate the TBK1/IRF3 as the normal membrane-bound STING [78]. This is because the transmembrane domain is reported to be critical for STING functioning as it is required for STING to relocate from the ER to the post-Golgi compartments during STING signaling. Any STING isoform without the TM domain is deemed non-functional [66]. Even though STING-β is non-functional on its own, it possesses a CTD domain that enables its binding to the STING, cGAMP and TBK1 as a result blocking them from interacting with their effectors (illustrated in Figure 6). Therefore, STING-β expression inversely correlates with IFN-I production [78]. 

### 4.6. Toll-like Receptor 4 (TLR-4)

TLR-4 is a member of the pathogen recognition receptor (PRR) family expressed in natural killer cells, macrophages, T cells, neutrophils, DCs and in cancer cells [79]. In lung cancer cells, TLR-4 activation may enhance immunosuppressive cytokine production that promotes apoptotic resistance [80]. TLR-4 is mainly activated by pathogen-associated molecular patterns (PAMPs) in a tumor microenvironment. It is essential for DC activation, maturation, differentiation and migration, and it is suggested to be responsible for the transformation from conventional to immunosuppressive regulatory DC in TME. TLR-4 promotes angiogenesis in the tumor microenvironment. Moreover, TLR-4 expressed on tumor-associated macrophages (TAM) is responsible for the migration of the TAMs into the TME [81]. 

**Figure 7:**
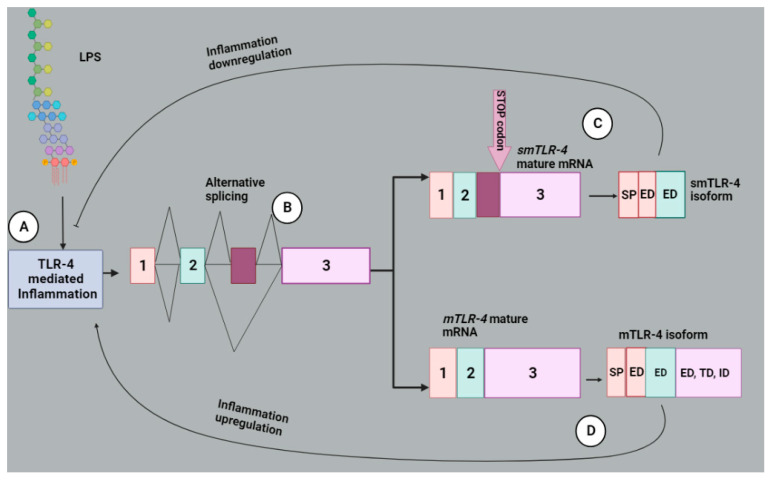
Negative feedback modulation by alternative splicing which reduces inflammation. (**A**) The LPS stimulates TLR-4, which then modulates inflammation, (**B**) triggering alternative splicing of *TLR-4* pre-mRNA. This splicing process generates two splice variants namely *smTLR-4* and *mTLR-4* of which undergoes translation to form smTLR-4 and mTLR-4 isoforms respectively with domains that could be similar to the ones in human TLR-4. (**C**) smTLR-4 inhibits inflammation via negative feedback inhibition whereas (**D**) mTLR-4 signaling upregulates inflammation. Image created with BioRender.com, accessed on 18 June 2023.

The human *TLR-4* gene consists of three exons with exon 1 encoding an SP and the section of the extracellular domain (ED). Then, exon 2 encodes another section of the ED, and exon 3 codes for the last section of the ED, TD and the cytoplasmic domain (CD) [82]. The extracellular domain is for the interaction with extracellular ligands, and the cytoplasmic domain is for interacting with TRIF and MyD88 [79]. Alternative splicing modulates a negative feedback mechanism that reduces inflammation. The stimulation of the TLR-4 signaling pathway by lipopolysaccharide (LPS) induces inflammation. On the other hand, LPS stimulation inhibits inflammation by altering the pre-mRNA splicing of genes that give rise to the proteins involved in the TLR-4 signaling pathway [83]. There is a pre-mRNA encoding soluble isoform of TLR-4 detected in mouse macrophages, which are induced during LPS stimulation that has an extra exon between exons 2 and 3 containing an in-frame STOP codon (Figure 7). This soluble isoform mediates a negative feedback mechanism of the TLR-4 pathway, as it reduces LPS-induced TNF-α production and NF-kB activation [83,84]. Another soluble TLR-4 isoform was detected in oral lichen planus (OLP) patients, which was shown to produce similar effects of negative regulation since cytokine production was inhibited in activated macrophages [85]. It is not known whether the dysregulated splicing of TLR-4 splicing contributes to the sTLR-4 secretion in humans [83]. sTLR-4 was detected in early-stage NSCLC patients and was suggested to be correlated with tumor metastasis and poor survival (Table 1) [40]. This isoform can combine with myeloid differentiation factor 2 (MyD-2) to form an sTLR4/MD-2 complex which can inhibit TLR-4 signaling by preventing the binding of the membrane-bound TLR-4 to its ligands [86].

### 4.7. Myeloid Differentiation Factor 88 (MYD88)

MyD88 is an inflammatory signaling adapter downstream of TLRs and IL-1R receptor families. It contains three domains, namely a C-terminal Toll or Interleukin-1 receptor (TIR) domain, an intermediate domain (ID) and an N-terminal death domain (DD). It activates IL-1R-associated kinase (IRAK) family kinases by linking it to IL-1R or TLR family members via its ID domain [41]. The activated IRAK family kinases result in several functional outputs, including the stimulation of NF-kB, MAPK and AP-1, causing MyD88 to be a central player in these inflammatory pathways. MyD88 signaling produces pro-inflammatory and IFN I cytokines [87,88]. MyD88 is highly expressed in colorectal cancer and plays a predominant role in promoting colorectal cancer cell proliferation, invasion and metastasis. The knocking down of MyD88 reduced the activity of NF-kB and AP-1 pathways that resulted in the inhibition of colorectal cancer progression [89]. 

**Figure 8:**
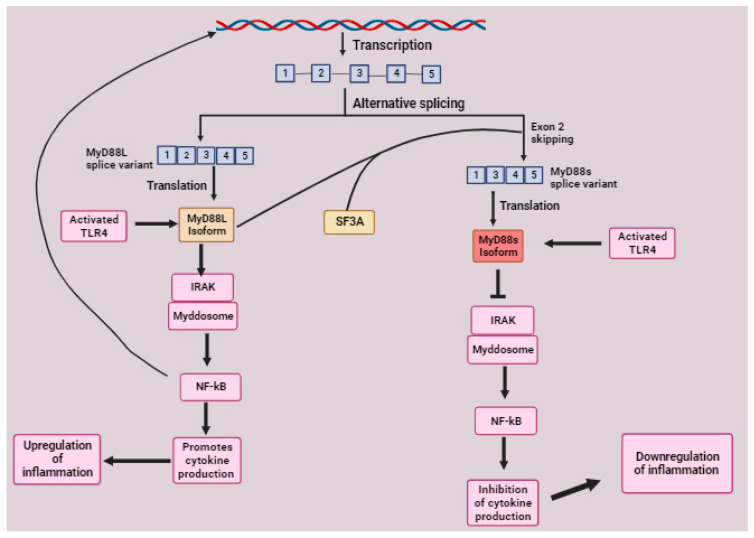
An illustration of the roles of MyD88L and MyD88s in TLR4-MyD88 signaling pathway. The MyD88 splicing generates two isoforms, namely the full-length MyD88L and the MyD88s lacking the ID domain crucial for linking TLR-4 to the IRAK-myddosome complex. MyD88L can activate the IRAK-myddosome complex by linking it to TLR-4 since it has the ID domain, followed by NF-kB activation promoting the transcription of pro-inflammatory cytokines, which upregulates inflammation. Moreover, NF-kB promotes the transcription of MyD88L, leading to the accumulation of many MyD88L isoforms that triggers its feedback inhibition by promoting the alternative splicing of MyD88s through the binding of MyD88L to the SF3A that causes exon 2 skipping to generate MyD88s lacking exon 2. The production of this ID domain lacking isoform is aimed at stopping the TLR-4/MyD88 signaling, followed by inhibition of cytokine production and downregulation of pro-inflammatory and subsequent inflammation. Image created with BioRender.com, accessed on 30 April 2023.

MyD88 has eight isoforms with different functions, including the full-length (MyD88L) and short MyD88 (MyD88s) isoforms [41]. The *MyD88L* splice variant has five exons, and the *MyD88s* splice variant has four exons due to the skipping of exon 2. This exon 2 encodes an ID essential for linking activated TLRs to the IRAK-containing Myddosome during signal transduction. Thus, MyD88s does not have this function and is regarded as a TLRs signaling inhibitor. This isoform is produced as a result of a negative feedback loop in mice macrophages to inhibit the TLR signaling and pro-inflammatory cytokine production. *MyD88s* is generated via the inhibition of SF3A and SF3B during alternative splicing in mice’s macrophages. MyD88L is responsible for the inhibition of the SF3A complex, which interacts with U2 snRNP during splicing. U2 snRNP is crucial for 3′ splice site recognition during the spliceosome assembly to the pre-mRNA. MyD88s splice variant is generated when the 3′ splice site intron 2 is used rather than the 3′ splice site in intron 1, resulting in the skipping of exon 2 [90]. It could be assumed that the binding of MyD88L to the SF3A interferes with the recognition of the 3′ splice site in intron 1 by U2 snRNP. The mechanisms of how the binding of MyD88L to SF3A influences the attachment of U2 snRNP to the branch point near the 3′ splice site at the end of intron 2 are not known (Figure 8). However, this negative feedback inhibition does not apply in B cell lymphomas because the prolonged TLR activation produces isoforms that enhance TLR and NF-kB signaling, such as the MyD88L rather than MyD88s (Table 1), which has an inhibitory effect [41]. Further studies are needed to understand the mechanisms of negative feedback inhibition by MyD88L and how the negative feedback loop does not apply in B cell lymphomas.

Methyltransferase-like 3 (METTL3) is another splicing factor involved in the modulation of alternative splicing of *MyD88s* in human dental pulp cells. An experimental study has proven that the depletion of the METTL3 leads to the production of MyD88s [91]. METTL3 is constantly overexpressed in CRC patients and is coupled with poor prognosis. It promotes CRC through the m6A-GLUT1-mTORC1 axis. However, METTL3 may have potential in the development of CRC-anti-cancer therapies since its combinatorial targeting with mTORC1 inhibited CRC growth [92]. Other MyD88 isoforms include MyD88N1 detected in liver, brain, heart and kidney tissues that lack the DD and the ID domains, as well as MyD88N2 detected only in brain tissue that does not code for any known functional domain [93].

### 4.8. CD44

CD44 is an 85–200 kDa transmembrane glycoprotein consisting of an extracellular domain, a transmembrane domain and an intracellular membrane domain. The extracellular domain senses stimuli and interacts with the external environment, and the transmembrane domain is responsible for providing an avenue for interaction with adaptor proteins and co-factors. An intracellular domain has a short and long tail configuration important for nuclear location and transcription mediation. There are several ligands interacting with CD44, namely hyaluronic acid (HA), collagens, osteopontin and matrix metalloproteinases (MMPs) [94]. CD44 is involved in mediating multiple signaling pathways that contribute to cancer cell division, angiogenesis, proliferation, invasion, and metabolic shift [95].

**Figure 9:**
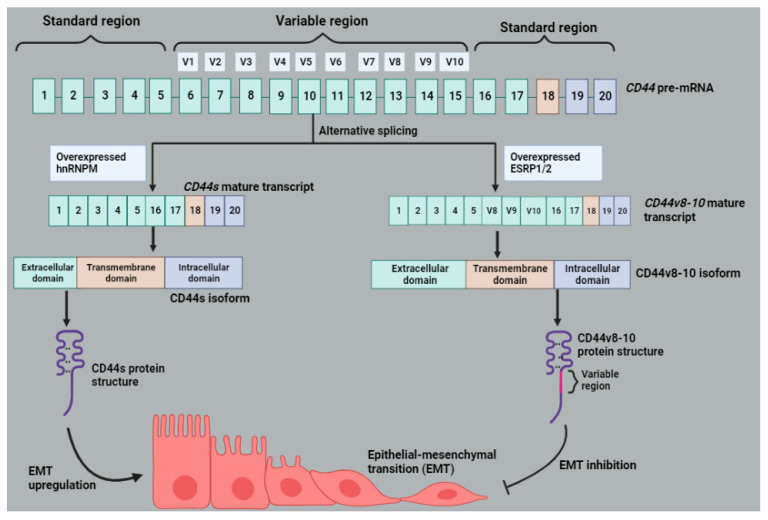
*CD44* splice variants, isoforms and their involvement in EMT. *CD44* pre-mRNA transcript generates two isoforms, namely CD44s and CD44v8-10. The formation of *CD44s* is regulated by the hnRNPM. CD44s is translated into CD44s isoform, which upregulates EMT. The formation of *CD44v8-10* variant is promoted by ESRP1 or 2. It undergoes translation forming CD44v8-10, which blocks EMT. Image created with BioRender.com, accessed on 28 June 2023.

There are two types of CD44 isoforms reported in the literature, namely the standard (CD44s) and the variant isoforms (CD44v) that are formed by alternative splicing of 10 variant exons [96]. Alternative splicing may generate an isoform switch to CD44s resulting in overexpression in hepatocellular carcinoma, which is crucial for a successful epithelial–mesenchymal transition (EMT) induced by TGF-β. An overexpression of hnRNPM regulates the switching of isoforms from CD44v to CD44s, whereas ESRP1 and 2 maintain the CD44v and block the EMT induced by TGF-β, snail, Twist, N-Cadherin or shRNA E-Cadherin, as shown in Figure 9. These splicing factors are also reported to compete for the cis-acting elements on the mRNA molecules. The high expression of CD44s in NSCLC corresponds with poor patient survival (Table 1) [42]. CD44v isoform is expressed in aggressive cancers than CD44s [97]. In CRC, the levels of CD44v containing exon 6 increase with the progression of cancer toward the metastatic stages (Table 1) [98]. This correlates with this study that indicated the absence of CD44v in non-metastatic brain tumors but abundant in metastatic brain tumors at about 86%. However, an inversely proportional relationship was observed in head and neck squamous cell carcinoma as CD44v6 levels diminish as the cancer progresses [97]. Furthermore, CD44v6 was shown to be associated with better patient survival post-operation in NSCLC [99]. whereas another study by Jiang and colleagues [42] reported poor NSCLC patient survival (Table 1). The overexpression of CD44V6 in CRC is associated with tumor invasiveness, colonization and metastasis [97,100]. CD44v8-10 is a major CD44 isoform expressed in GC cells. Its overexpression in GC cells is associated with disease initiation in immunocompromised mice and is regarded as a potential cell surface marker of GCSCs [101]. 

### 4.9. Fibroblast Growth Factor Receptor 2 (FGFR2)

**Figure 10:**
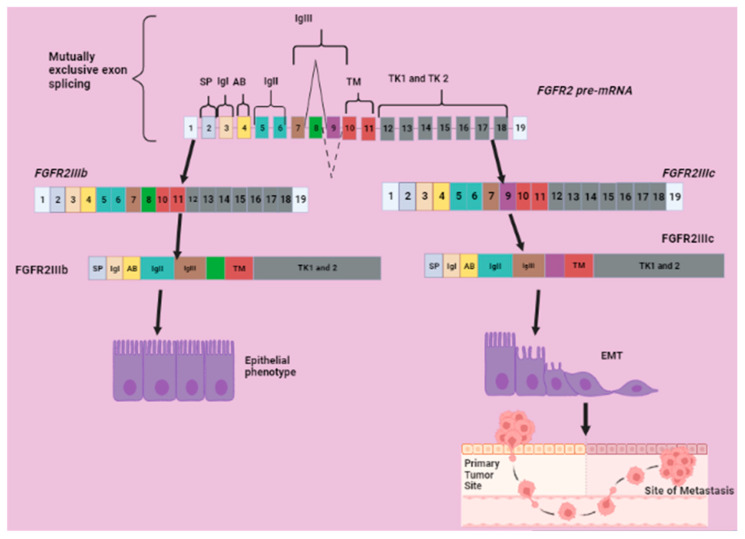
*FGFR2* splice variants, isoforms and their involvement in EMT-associated metastasis. Mutually exclusive splicing of *FGFR2* generates two splice variants, namely *FGFR2IIIb* containing exon 8 and *FGFR2IIIc* containing exon 9. *FGFR2IIIb* is translated into FGFR2IIIb, which upregulates the epithelial phenotype. *FGFR2IIIc* is translated into FGFR2IIIc isoform, which promotes EMT-associated metastasis. Image created with BioRender.com, accessed on 28 June 2023.

FGFR2 is a member of the tyrosine kinase receptor family [102] comprising of three immunoglobulin (Ig)-like extracellular domains, two of which are involved in ligand binding, a transmembrane domain, tyrosine kinase, and a C-terminal tail containing multiple autophosphorylation sites [103]. The FGFR signaling pathway regulates organogenesis and homeostasis under normal physiological conditions, and the dysregulation of this pathway results in tumorigenesis [102]. FGFR2 signaling is linked to downstream pathways, including PIK3-AKT-mTOR, JAK-STAT and RAS-MAPK, associated with promoting tumor growth, invasion, EMT and angiogenesis [104]. Gona [105] has indicated that the mouse *FGFR2* gene consists of 19 exons, of which exons 2, 3 and 4 encode SP, the first immunoglobulin-like domain (IgI) and acid box (AB), respectively. Exons 5 and 6 encode the second immunoglobulin-like domain (IgII), exons 7, 8 and 9 encodes the third immunoglobulin-like domain (IgIII), and exons 10 and 11 encode the TM domain. Exons 12 to 18 encode the split tyrosine kinase domain 1 and 2 (TK1 and TK2) (Figure 10).

The alternative splicing of *FGFR2* generates isoforms with dissimilar Ig-like domains and varying ligand binding affinities [103] and distinct activation of downstream signaling pathways [45]. FGFR2IIIb isoform binds FGF1, 3, 7, 10 and 22 with high affinity, whereas FGFR2IIIc is a receptor for FGF1, 2, 4, 6, 8 and 18 [106]. FGFR2IIIb is associated with the epithelial cell phenotype, whereas FGFR2IIIc is associated with the mesenchymal cell phenotype (shown in Figure 10) [107]. ESRPs regulate the alternative splicing of *FGFR2* by binding to the cis-acting intronic elements between exon IIIb (8) and IIIc (9) to promote the isoform switch towards FGFR2IIIb, which favors the epithelial phenotype [108]. An aberrant expression of FGFR2IIIc triggers EMT in epithelial cells and is tumor promoting, whereas FGFRIIIb is tumor suppressive [109]. The expression of FGFR2IIIc in clear cell renal cell carcinoma (ccRCC) was found to be associated with poor clinical outcomes [108]. Furthermore, this isoform seems to be antagonizing the functions of FGFR2IIIb in keratinocyte differentiation because FGFR2IIIb modulates the differentiation of keratinocytes through the sequential inclusion of PKCδ and PKCα signaling. However, the aberrant expression of the FGFR2IIIc isoform altered the FGFR ligand specificity, and this resulted in an impairment of keratinocyte differentiation, early oncogenic features and an EMT [110]. The anti-tumor properties of FGFR2IIIb were shown in HCC as the downregulation of the expression of FGFR2IIIb in HCC correlated with invasion and high tumor stage. Then, the re-expression of FGFR2IIIb in the same cells reduced their proliferation and migration in vitro [7]. Moreover, this isoform attenuated tumor growth in the salivary gland, bladder and malignant prostate cancer cell lines [45] and also suppressed EMT-associated metastasis in pancreatic ductal adenocarcinoma (PDAC) [111]. However, FGFR2IIIb was shown to be associated with poor prognosis in pancreatic and lung cancer. Therefore, FGFR2IIIb might be having both pro-and anti-tumor effects [45].

## 5. Therapeutic Interventions

CD44 monoclonal antibody, namely A3D8, binds to CD44s and promotes apoptosis in acute myeloid leukemia cells. Sulfasalazine engages with CD44v to decrease the survival of CD44v+ human gastric cancer stem cells in vivo and in vitro. The combination of curcumin and epigallocatechin gallate suppressed breast CSCs by inhibiting STAT3 and NF-κB signaling pathways. The combination of 5-fluorouracil with Silibinin inhibited CD44v6+ subpopulation cell proliferation in human colon carcinoma cells [112]. Bemarituzumab, which is a selective monoclonal antibody for FGFR2IIIb, is currently being investigated in a phase III randomized trial for advanced GC patients. It targets the FGFR2IIIb receptor, particularly the third immunoglobulin section of the FGFR2IIIb receptor as a result blocking FGF ligand binding. This inhibits sequential downstream tumor-promoting signaling, such as receptor dimerization and FGFR2IIIb phosphorylation [113]. Alofanib (RPT835) is an FGFR2 inhibitor with a potent activity that was observed in gastric, lung, breast and ovarian cancer models in pre-clinical studies [114]. A small phase Ib study of alofanib on patients with metastatic gastric adenocarcinoma reported no dose-limiting toxicities and the recommended phase II dose [113]. Alofanib is a small-molecule inhibitor of FGFR2IIIc and IIIb isoforms with half-maximal inhibitory concentration (IC_50_) < 10 nM [113], which binds to the non-active site of FGFR2 extracellular domain [114].

sCTLA-4 blockade with isoform-specific antibodies has been proven to induce productive antitumor responses in murine cancer models [33,115]. However, the immune checkpoint blockades antibodies currently available clinically cannot distinguish between membrane-bound and soluble isoforms, while the soluble isoforms contribute to immunosuppression [115]. Based on the immunotherapy’s low efficacy, adverse events and drug resistance reported [116], immune checkpoint blockades should be improved to be isoform-specific to avoid immunosuppression or interference by the other isoforms of the targeted protein [117].

There are compounds used for aberrant splicing-related cancers, including GEX1 [118], spliceostatins, sudemycins, FD-895, E7107 and FR901464, which all act directly on the core spliceosomal component SF3B1, of which accounts for 10% splicing events [119]. The use of protein arginine methyltransferases (PRMTs) is an attractive approach to targeting splicing. They catalyze arginine dimethylation of various substrates, including RBPs and splicing factors [118]. GSK3368715 and PF06939999, GSK3326595 and JNJ-64619178 are type I and PRMT5 inhibitors currently in clinical trials, respectively. The depletion or chemical inhibition of PRMT5 inhibits splicing and produces anti-cancer effects in various cancers [120].

Targeting the core components of the spliceosome disrupts the early stages of spliceosome assembly and may result in various nonspecific and toxic effects. Hence, it was suggested that specific spliceosome components and splicing factors associated with aberrant splicing events should be targeted [121]. Therefore, detailed splicing mechanisms of these immunomodulatory proteins should be further explored for application in the development of therapies targeting specific splicing factors associated with their aberrant splicing.

## 6. Conclusions and Future Perfectives

**Figure 11:**
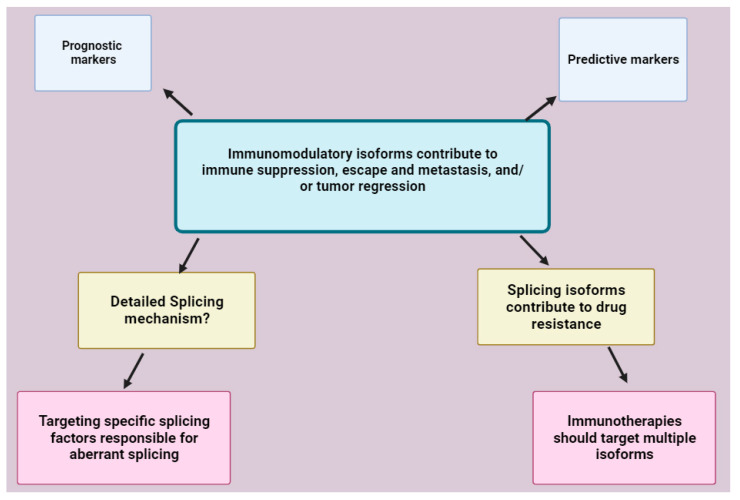
Conclusion illustration. Immunomodulatory protein isoforms contribute to immune suppression, escape and metastasis and, in some instances, may also contribute to tumor regression. They have shown a potential for use as predictive or prognostic markers. Certain isoforms such as the soluble isoforms have drug (antibody) resistance effects. Therefore, immunotherapies should target all the isoforms associated with a protein of interest. In addition to the use of antibodies, the production of immunomodulatory isoforms can be targeted at a splicing level. This will require the use of therapies targeting specific splicing factors or components to avoid non-specificity and toxicity. Therefore, the detailed alternative splicing mechanisms of these immunomodulatory genes require further studies for the knowledge to be applied in the development of such therapies. Image created with BioRender.com, accessed on 30 June 2023.

Alternative splicing is a complex post-transcriptional modification process involving several regulatory factors. In the TME, alternative splicing may act as a double-edged sword, as it produces isoforms that generate an immune response favoring both tumor progression and regression. The net immune response generated by the immunomodulatory proteins in the TME depends on the interaction between receptors and their ligands whether soluble or membrane bound. The full-length isoforms, which are membrane bound, mostly have the original function of the immunomodulatory proteins, whereas soluble isoforms may have different or opposite functions. The soluble isoforms are mostly immunosuppressive and favor cancer progression by facilitating tumor cell proliferation, invasion, and metastasis. However, soluble isoform, namely PD-1, may stimulate an anti-tumor immune response. Immunotherapeutic interventions should aim at counteracting the isoforms that negatively impact the immune response and enhance the activity of isoforms that positively regulate the immune response. The soluble isoforms are mostly used as prognostic and predictive markers in various cancer types. The presence of soluble isoforms generates drug resistance as they may act as decoys and bind the drugs for example in PD-L1 blockade therapies. Therefore, the presence of the isoforms associated with target proteins needs to be considered when designing therapies. The use of compounds targeting the specific components of the spliceosome and splicing factors is ideal rather than the ones that target core components of the spliceosome due to non-specificity and toxicities that may arise. These molecules have been demonstrated to show promising anti-cancer effects on other splicing factors. However, there is limited information on splicing factors associated with the aberrant splicing of immunomodulatory proteins. Therefore, their detailed splicing mechanisms need to be explored to reveal the specific spliceosome components and splicing factors associated with their aberrant splicing, which can then be applied in the development of therapies (Figure 11). 

## Figures and Tables

**Table 1:** Immunomodulatory isoform expression levels in various tumor types and their association with clinical outcomes.

Isoform	Tumor Type	Expression Levels in Tumor Tissue	Expression Levels in Normal Tissue	Clinical Results	References
sCTLA-4	Melanoma	High	Unknown	Associated with drug response and better patient survival	[31]
	Glioma	High	Low	Shorter survival	[34]
PD-1Δex3	Breast (TNBC)	High	Low	Associated with high tumor stage	[36]
PD-L1-1	Melanoma	High	Low	Melanoma progression	[37]
PD-L1-3					
PD-L1-9					
PD-L1-242	NSCLC	High	Low	Drug resistance	[38]
PD-L1-229					
sHLA-G	Melanoma	High	Low	Advanced disease stage and tumor load	[39]
sTLR-4	NSCLC	High	Low	Tumor metastasis and poor survival	[40]
MyD88s	B cell lymphoma	Comparable	Comparable	Unknown	[41]
MyD88L		High	Low		
CD44V6	NSCLC	High	Unknown	Poor patient survival	[42]
CD44s	NSCLC	High	Unknown	Poor patient survival	
CD44V8-10	Breast cancer (basal-like subtype)	Unknown	Unknown	Better overall 10-year patient survival	[43]
	GC	High	Low	Unknown	[44]
FGFR2IIIc	GC	Low	High	Low expression correlates with better patient survival	[45]
FGFR2IIIb	GC	High	Low	Unknown	[45]

## Data Availability

Not applicable.
